# Vitamin A-retinoid signaling in pulmonary development and disease

**DOI:** 10.1186/s40348-016-0054-6

**Published:** 2016-08-01

**Authors:** Hector A. Marquez, Wellington V. Cardoso

**Affiliations:** 1 Pulmonary Center, Boston University School of Medicine, Boston, MA 02118 USA; 2 Department of Medicine, Columbia Center for Human Development, Columbia University Medical Center, New York, NY 10032 USA

**Keywords:** Retinoic acid, Vitamin A, Lung development, Airway hyperresponsiveness, Smooth muscle, Asthma, Developmental pathways

## Abstract

Retinoic acid (RA), the active form of vitamin A, regulates key developmental processes in multiple organs. In the developing lung, RA is crucial for normal growth and differentiation of airways. Disruption in RA signaling or vitamin A deficiency (VAD) has been linked to aberrant development of the lung including alterations in the airway smooth muscle (SM) differentiation, development, and function. These alterations have been linked to disease states including asthma in both human and animal models.

## Introduction: retinoic acid, a crucial signal for formation of the embryonic lung

Classic studies in multiple species, including hamsters, rats, and mice, have shown major pleiotropic effects of maternal dietary vitamin A deprivation in embryonic development. The dramatic developmental defects in the cardiovascular, digestive, and respiratory tract support a major role for retinoic acid (RA) signaling in regulating key events during organogenesis.

The developing lung is particularly sensitive to changes in the levels of RA; vitamin A deficiency is known to result in lung hypoplasia and, when most severe, lung agenesis, the inability to form primordial lung buds [1]. Components of the RA pathway include enzymes that synthesize and metabolize RA, nuclear receptors (retinoic acid receptors (RARs) and retinoid X receptor (RXRs)) that transduce retinoid signaling, retinoid-binding proteins, and a number of co-activators and repressors that modulate retinoid signaling. These components are all expressed in the foregut as early as at the onset of lung development [2]. The presence in the foregut of strong *Aldh1a2* signals, which encodes an enzyme essential to generate the RA, suggests high RA synthesis. Analysis of a RARElacZ transgene reporter confirms the extensive local RAR activation at the time of primary lung bud formation [3]. Disruption of *Aldh1a2*, in mouse mutants, reproduces the lung agenesis phenotype reported in the severe vitamin A deficiency models.

While no lung buds form in the RA-deficient foregut endoderm lung, progenitor cells are still specified at the prospective lung field. This implies that, although required for formation of the lung bud primordium, RA signaling is dispensable for specification of lung progenitor cells [4–6]. Accumulated evidence also supports a role of RA signaling in the initial steps of airway morphogenesis; RAR-a:RAR-b2 double knockout mice show left lung agenesis and right lung hypoplasia [7].

**Fig. 1:**
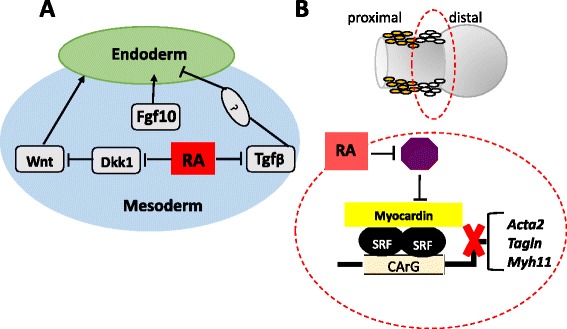
Retinoid regulation of early lung development. **a** At the onset of lung development, mesodermal RA signaling suppresses Dkk1 to allow induction of Wnt pathway and inhibits Tgf-beta signaling to de-repress Fgf10 expression. Thus, RA coordinately controls proper Fgf10 levels to activate Fgf signaling to the foregut endoderm and form the lung primordium. RA regulation of Tgf-beta effects in endoderm has also been suggested. **b** During airway formation, mesodermal (mesenchymal) RA signaling restricts SM gene expression (Acta2, Myh11, Tagln) in distal lung, preventing ectopic and excessive SM differentiation while airways are branching. RA may potentially inhibit a key activator of SM transcription or induce a transcriptional repressor to control SM gene expression (diagram: myocardin, serum response factor, CArG DNA binding sites at SM gene promoters). Modified from Chen et al. [14, 15]

Genome-wide screen of RA targets in the foregut at the onset of lung development has provided important insights into the RA-dependent events required for lung formation. RA influences expression of a large number of mesenchymal genes involved in lung morphogenesis. A major conclusion that emerged from these studies was that endogenous RA coordinately controls Wnt, Tgf-beta, and Fgf, three pathways crucial for formation of lung buds (Fig. 1a). As confirmed by both in vitro and in vivo mouse genetic models, disruption of endogenous RA signaling leads to hyper-activation of Tgf-beta signaling throughout the foregut region where the lung arises. Hyperactive Tgf-beta dramatically inhibits local expression of Fgf10 necessary for induction of lung buds. Furthermore, RA deficiency results in widespread expression of the Wnt inhibitor *Dkk1* in the foregut mesoderm. The Dkk1-mediated repression of Wnt signaling further contributes to downregulate Fgf10 expression locally, leading to inability to expand the initial pool of lung progenitor cells and form the lung primordium [8, 9]. These studies establish RA as key signal in balancing the activity of Wnt and Tgf-beta in the foregut mesoderm to control the proper levels of Fgf10, a factor that is ultimately required for formation of the lung primordium. This mechanism likely represents the molecular basis of the lung agenesis classically described in the models of vitamin A deficiency.

## Prenatal vitamin A-RA signaling influences the program of smooth muscle differentiation in developing airways

The developing lung mesenchyme gives rise to a number of derivatives including endothelium, cartilage rings, interstitial fibroblasts, and vascular and airway smooth muscle. Smooth muscle (SM) represents a major component of the vascular and airway compartment [10]. Airway SM originates early in the developing lung mesenchyme, during formation of the bronchial tree, through a mechanism that is distinct from that of the lung vascular musculature. Mature SM is not terminally differentiated and retains a certain degree of plasticity allowing to assume two distinct phenotypes: a more proliferative and able to synthesize extracellular matrix (ECM) components (synthetic) or a more differentiated, expressing preferentially more mature SM markers. Phenotype switch has been associated with repair in response to local cues but can be aberrantly present in chronic pulmonary conditions, such as asthma [11–13].

A link between RA and SM at the onset of lung development was first suspected from the analysis of genome-wide of foreguts, which showed an intriguing upregulation of genes associated with SM differentiation in RA-deficient foreguts compared to the RA-sufficient controls. These genes included alpha SM actin (Acta2), cysteine- and glycine-rich proteins 1 and 2 (Csrp1 and Csrp2), transgelin (Tagln), myosin, heavy chain 11, smooth muscle (Myh11). Subsequent analysis showed that expression of these genes was also markedly enhanced in lung explants or lung mesenchymal cells in which RA signaling was disrupted by RAR antagonist [14]. Confirmation of this RA-SM regulation in vivo was provided by evidence that restricting maternal vitamin A dietary intake during initiation of airway development resulted in aberrant overly differentiated and ectopic distribution of SM in these airways.

Interestingly, analysis of RA reporter mouse (RARElacZ transgene) shows that, when airways are forming and branching, RA is very active in the mesenchyme associated with the stalks of newly formed buds, sites of initiation of a SM program in developing airways. The decreasing RA reporter signals in proximal airways where SM is already formed and the aberrant SM phenotype of vitamin A-RA deficiency strongly suggest that endogenous RA represses SM differentiation. A mechanism has been proposed in which endogenous RA temporarily inhibits the development of SM in airways in areas that are still branching, preventing precocious and excessive formation of SM cells. Indeed, sites of RA activation are also site of activation of pathways involved in SM differentiation, as well illustrated by Tgf-beta, a known target of RA signaling [15] (Fig. 1b). It is also of interest that RA deficiency does not seem to affect the SM program in blood vessels, arguing for distinct mechanisms and molecular regulation of SM differentiation in different structures.

## Vitamin A-RA and postnatal airway disease

A number of epidemiological studies in children and adult subjects have linked vitamin A status with chronic airway diseases. There have been multiple studies linking low serum vitamin A levels to prevalence and severity of asthma. In populations in which vitamin A deficiency is prevalent, studies have shown that children with asthma have lower vitamin A levels than those who do not have asthma [16]. On multivariate analysis, severity of disease was highly correlated with serum vitamin A levels. Studies have also linked severity of vitamin A deficiency with severity of wheezing in infants [17]. Interestingly, a study in a well-nourished population of Japanese children showed mean serum vitamin A concentration significantly lower in those asthmatic than in non-asthmatic control subjects [18]. Decreased FEV1 has been reported in both children and adolescents with vitamin A deficiency [19]. Supplementation with beta-carotene has also been shown to have a protective effect against exercise-induced asthma [20].

In the adult lung, vitamin A deficiency results in marked changes in the respiratory epithelium, including necrotizing tracheobronchiolitis and squamous metaplasias (transformation of a respiratory pseudostratifed into a skin-like stratified epithelium). Once vitamin A levels were restored to normality, these changes were reversed. These abnormalities have been also observed in ventilated infants with chronic neonatal lung injury. Premature infants were shown to have lower concentrations of plasma vitamin A, raising the possibility that vitamin A deficiency in preterm infants may contribute to increased risk of developing chronic lung disease. A series of nine trials have been recently reviewed to determine the impact of vitamin A supplementation in preventing mortality and short- and long-term morbidities in very low birth weight infants [21]. A meta-analysis of these data [21] showed that at 36 weeks of age, there was a significant reduction in oxygen use in the preterm infants treated with vitamin A supplementation compared to the control (placebo) group, although only a trend towards reduction of death or chronic lung disease was observed. At 1 month of age, only one of the nine trials showed a significant reduction of death or chronic lung disease in the infants of the vitamin A group. The majority of these studies used an intramuscular regimen of a “standard” dose vs a higher dose of vitamin A. There was no significant difference in the outcomes based on standard vs higher doses of vitamin A. No adverse effects due to vitamin A supplementation were reported in these trials [21].

Allergy and pro-inflammatory conditions can play an important role in the pathogenesis of pulmonary disorders that lead to airway hyperresponsiveness and asthma. However, hyperresponsiveness may well occur in the absence of inflammation. For example, vitamin A-deficient adult rats exposed to aerosolized methacholine have an increase in total pulmonary resistance compared to control rats. Analysis of different functional parameters revealed that this hyperresponsiveness resulted from diminished muscarinic receptor-2 (M2R) function in the vagal pre-junctional terminals [22]. The effect could be partially reversed by restoring normal RA levels. Thus, multiple mechanisms potentially contribute to link abnormal airway function with RA-mediated responses.

## RA-mediated events and the developmental origins of airway disease

There is accumulated evidence that fetal exposure to adverse conditions, such as nutritional imbalances, xenobiotics, or physical insults at critical stages, can leave a “memory” or a structural defect carried throughout life. These changes can have a major impact on how individuals respond to environmental stimuli postnatally and thus influence susceptibility to disease. The idea that changes in vitamin A-RA status during fetal life can alter structural and mechanical properties of the lung in adult life has been suggested in few studies. Mild maternal vitamin A deficiency in pregnant rats resulted in changes in the composition of the extracellular matrix of the lung in adult offspring; these mice showed increased collagen and decreased elastin deposition in the lungs. [23]. Potential changes in elastogenesis during fetal development can result in near-permanent defects in the adult lung [24].

A study in a population with chronic vitamin A deficiency showed that maternal vitamin A deficiency has a negative impact in postnatal lung function of the offspring with reduced FEV1, FVC in childhood. These effects can be prevented or alleviated by proper vitamin A supplementation during gestation [25]. The impact of prenatal disruption of RA signaling in adult airway structure and function has been well demonstrated using a model of vitamin A deficiency in mice [14]. Aberrant airway SM phenotype with increased and ectopic expression of SM markers is found in embryos from mothers exposed to a short developmental window of vitamin A deficiency. Remarkably, if returned to normal vitamin A diet later in gestation and throughout their postnatal life, adult mice show a markedly thick airway SM layer. Pulmonary function tests reveal that mice exposed to prenatal RA deficiency have increased airway resistance when challenged with spasmogenic stimuli, as compared to controls. These observations strongly support the idea that prenatal vitamin A deficiency predisposes to airway hyperreactivity. The functional-structural changes in the airways in the absence of inflammation remind us of the multifactorial origin of conditions that lead to hyperresponsiveness and asthma, indicating that additional components should not be overlooked in the pathogenesis of these conditions.

## Conclusions

Overall, the observations here underscore the importance of dietary vitamin A/retinoids, a significant challenge in developing countries. Vitamin A deficiency is a significant public health problem in more than half of all world’s countries affecting particularly children and women during pregnancy in developing nations. Retinoid signaling has a major impact in the development and maintenance of multiple organ systems. In spite of the major recent advances in retinoid biology, still there are many questions about the mechanism and cellular targets of this pathway. Circulating levels of vitamin A (retinol), retinyl esters, or RA do not necessarily reflect the retinoid status of target cells, since activation of RA signaling depends on a complex interplay of the components of this pathway, including the presence of RA-synthesizing enzymes, RA receptors (RAR, RXR), co-activators/co-repressors, and RA-metabolizing enzymes. The efficiency of this system is subjected to the interference by multiple environmental adverse factors, including cigarette smoke and alcohol, which can disrupt crucial RA-mediated developmental events. Thus, environmental factors that interfere with expression or activation of any of these components may have a profound effect in the net activity of RA signaling, which may vary in the multiple cell types of a particular microenvironment. Future studies addressing this complexity are likely to open major venues for discovery and development of strategies for the use of retinoid as therapeutical targets.

## Abbreviations

RA, retinoic acid; RARs, retinoic acid receptors; RXR, retinoid X receptor; SM, smooth muscle; VAD, vitamin A deficiency
